# Left ventricular systolic function evaluated by strain echocardiography and relationship with mortality in patients with severe sepsis or septic shock: a systematic review and meta-analysis

**DOI:** 10.1186/s13054-018-2113-y

**Published:** 2018-08-04

**Authors:** F. Sanfilippo, C. Corredor, N. Fletcher, L. Tritapepe, F. L. Lorini, A. Arcadipane, A. Vieillard-Baron, M. Cecconi

**Affiliations:** 1 Department of Anaesthesia and Intensive Care, IRCCS-ISMETT (Istituto Mediterraneo per i Trapianti e Terapie ad alta specializzazione), Via Tricomi 5, 90127 Palermo, Italy; 2 Department of Perioperative Medicine, Bart’s Heart Centre St. Bartholomew’s Hospital, W. Smithfield, London, UK; 3 Department of Anaesthesia and Critical Care, St Georges University Hospitals NHS Trust, Blackshaw Road, London, SW170QT UK; 4 Department of Cardiovascular, Respiratory, Nephrological, Anaesthetic and Geriatric Sciences, Sapienza University of Rome, Rome, Italy; 5 Department of Anaesthesia and Intensive Care, Papa Giovanni XXIII Hospital, Bergamo, Italy; 6 Assistance Publique-Hopitaux de Paris, University Hospital Ambroise Paré, Intensive Care Unit, Section Thorax-Vascular Disease-Abdomen-Metabolism, 92100 Boulogne-Billancourt, France; 7 INSERM U-1018, CESP, Team 5 (EpReC, Renal and Cardiovascular Epidemiology), Universite’ Versailles Saint Quentin en Yvelines, 94807 Villejuif, France; 8 Humanitas Clinical and Research Center, Via A. Manzoni 56, 20089 Rozzano – Milan, Italy; 9 Humanitas University, Department of Biomedical Sciences, Via Rita Levi Montalcini 4, 20090 Pieve Emanuele – Milan, Italy

**Keywords:** Global longitudinal strain, Intensive care, Left ventricular ejection fraction, Speckle tracking, Systolic dysfunction

## Abstract

**Background:**

Sepsis-induced myocardial dysfunction is associated with poor outcomes, but traditional measurements of systolic function such as left ventricular ejection fraction (LVEF) do not directly correlate with prognosis. Global longitudinal strain (GLS) utilizing speckle-tracking echocardiography (STE) could be a better marker of intrinsic left ventricular (LV) function, reflecting myocardial deformation rather than displacement and volume changes. We sought to investigate the prognostic value of GLS in patients with sepsis and/or septic shock.

**Methods:**

We conducted a systematic review (PubMed and Embase up to 26 October 2017) and meta-analysis to investigate the association between GLS and mortality at longest follow up in patients with severe sepsis and/or septic shock. In the primary analysis, we included studies reporting transthoracic echocardiography data on GLS according to mortality. A secondary analysis evaluated the association between LVEF and mortality including data from studies reporting GLS.

**Results:**

We included eight studies in the primary analysis with a total of 794 patients (survival 68%, *n* = 540). We found a significant association between worse LV function and GLS values and mortality: standard mean difference (SMD) − 0.26; 95% confidence interval (CI) − 0.47, − 0.04; *p* = 0.02 (low heterogeneity, *I*^2^ = 43%). No significant association was found between LVEF and mortality in the same population of patients (eight studies; SMD, 0.02; 95% CI − 0.14, 0.17; *p* = 0.83; no heterogeneity, *I*^2^ = 3%).

**Conclusions:**

Worse GLS (less negative) values are associated with higher mortality in patients with severe sepsis or septic shock, while such association is not valid for LVEF. More critical care research is warranted to confirm the better ability of STE in demonstrating underlying intrinsic myocardial disease compared to LVEF.

**Electronic supplementary material:**

The online version of this article (10.1186/s13054-018-2113-y) contains supplementary material, which is available to authorized users.

## Background

A recent expert consensus updated the definition of sepsis as “life-threatening organ dysfunction caused by a dysregulated host response to infection” [1]. The response to the infective process can cause profound hemodynamic alterations, leading to organ dysfunction and accounting for high mortality and morbidity [2, 3]. The most severe manifestation of sepsis is septic shock, a condition characterized not only by vasoplegia but that can also present with myocardial depression. Septic cardiomyopathy is the widely used term for myocardial involvement in sepsis [4], and can present with left and/or right ventricular (LV and/or RV) impairment [5]. The majority of patients with sepsis and/or septic shock have underlying cardiac dysfunction as demonstrated by a post-mortem necropsy study that showed myocardial injury in more than half of patients with sepsis and/or septic shock [6], although such patients do not always exhibit signs and symptoms of myocardial ischemia [7].

While there is growing evidence of an association between LV diastolic dysfunction and mortality [8, 9], the influence of LV systolic dysfunction (LVSD) is less clear. Initial evidence suggested that mean LV ejection fraction (LVEF, the most commonly used index to define LVSD) is paradoxically higher in non-survivors of septic shock [10], although this association has not been subsequently confirmed [11]. Finally, a meta-analysis showed no association between LVEF and mortality in a septic population [12], and this has been confirmed by subsequent work [8].

Echocardiography is currently suggested as part of the first-line approach in the evaluation of patients with shock [13], but the use of conventional indexes of systolic function in patients with sepsis and/or septic shock may not accurately reflect the true systolic function. Traditional estimation of LVSD is problematic in sepsis since LVEF is based on significant geometric assumptions and is also highly dependent on loading conditions. Preload is highly variable according to fluid and vasoactive drug resuscitation, degree of endothelial insult and vascular leak. Afterload variations in sepsis (vasoplegia and vasoconstrictor use) causes changes in LVEF that are not necessarily related to true variations in intrinsic myocardial contractility [14].

**Fig. 1:**
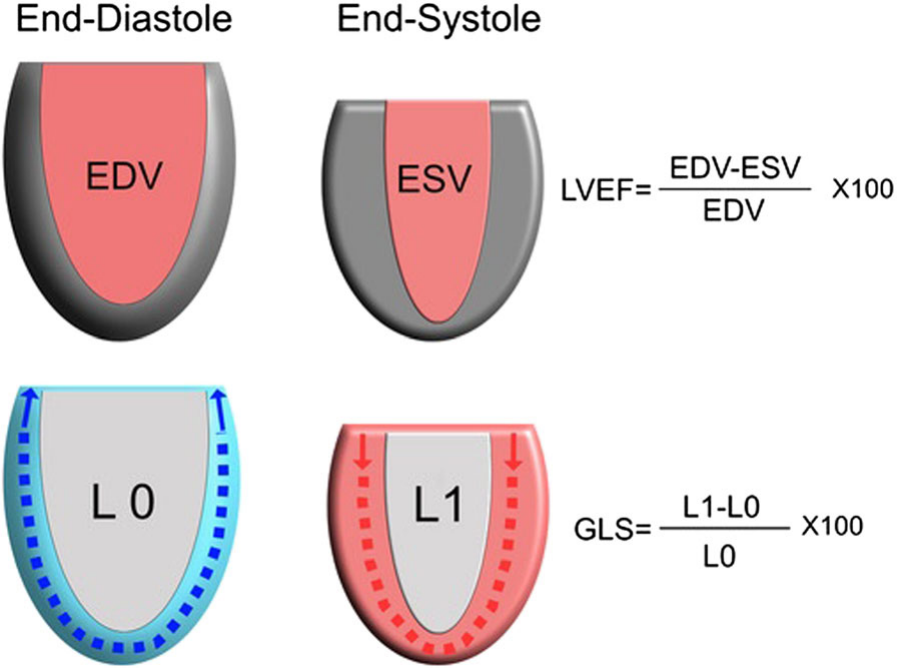
Illustration of differences between left ventricular ejection fraction (LVEF) and global longitudinal strain (GLS). EDV, end-diastolic volume; ESV, end-systolic volume; L, length; L0, total longitudinal length of the left ventricular (LV) border in diastole; L1, total longitudinal length of the LV border in systole

An alternative echocardiographic modality, speckle-tracking echocardiography (STE) is emerging as a better marker of intrinsic LV function [15]. It was first described in 2004 as an angle-independent non-Doppler method [16], based on the generation of ultrasound echoes (“speckles”) representing discrete myocardial areas tracked throughout the cardiac cycle [17]. Strain represents the difference between the final length of each segment relative to its resting length and can be measured in different planes: longitudinal (from base to apex), radial (inward short axis), and circumferential (rotational short axis). Its assessment is performed during bedside echocardiography, and global longitudinal strain (GLS) is the most commonly reported strain measure, representing the ratio of the maximal change in the myocardial longitudinal length in systole to the original length in diastole. In short, more negative values of GLS indicate better LV systolic function. The conceptual difference between assessment of myocardial function with GLS or LVEF is illustrated in Fig. 1.

In this systematic review and meta-analysis we aim to investigate the association between values of strain and mortality in patients with severe sepsis and/or septic shock. We hypothesize that GLS, but not LVEF values, are associated with mortality in patients suffering from severe sepsis and/or septic shock.

## Methods

This systematic review and meta-analysis was performed in accordance with preferred reporting items for systematic reviews and meta-analyses (PRISMA) guidelines [18]. The review was registered with the international prospective register of systematic reviews (PROSPERO, number CRD 42016041712).

### Eligibility criteria

Since the definition of sepsis and septic shock changed only recently, we included in our meta-analysis prospective studies providing data on mortality of patients with severe sepsis and/or septic shock, as defined by the previously widely accepted international consensus [19]. Studies with non-prospective design were included in sensitivity analyses.

Studies were included in the analysis if they provided values for strain echocardiography in survivors and non-survivors in a population including patients with severe sepsis and/or septic shock. In the event of studies reporting only the overall population strain values, we contacted the authors to increase data availability. Inclusion criteria were pre-specified using the patient, population or problem, intervention, comparison, outcomes and study design (or setting) (PICOS) framework (Table 1). Pediatric populations were excluded to minimize heterogeneity. Only case series reporting LVSD data and outcomes from at least 10 patients were included.

**Table 1:** “PICOS” approach for selecting clinical studies in the systematic search

PICOS	Characteristics of clinical studies included for the qualitative synthesis and meta-analysis
1. Participants	Adult patients with severe sepsis and/or septic shock
2. Intervention	Strain echocardiographic assessment with TTE, conducted within the first 48 h from diagnosis
3. Comparison	Primary: comparison of GLS values between survivors and non-survivors Secondary: comparison of LVEF between survivors and non-survivors (only studies providing GLS) Sensitivity: leave-one-out-at-a-time, excluding studies with high risk of bias, including studies performing TTE within 1 week; including retrospective and case-control studies
4. Outcomes	Mortality (at longest follow-up available)
5. Study design	Prospective clinical studies

*PICOS* patient, population or problem, intervention, comparison, outcomes and study design (or setting), *GLS* global longitudinal strain, *LVEF* left ventricular ejection fraction, *TTE* trans-thoracic echocardiography

### Identification of studies

A systematic search of the electronic databases - MEDLINE (PubMed) and EMBASE - was performed through the National Health Service (NHS) Healthcare Databases Advanced Search. Relevant titles were also identified by hand-searching reviews on the topic and exploring the list of the references of the selected papers. There was no date restriction and only articles published in English, Spanish, French, German or Italian were considered. Duplicates were filtered through automated function and then manually searched. Titles retrieved from EMBASE as conference abstracts were considered only if published after October 2015 to allow a reasonable time for adequate peer review. The last search update was on 26 October 2017.

The findings of two search-term groups were combined: the items “bacteraemia”, “bacteremia”, “respiratory distress syndrome”, “sepsis”, “septic shock”, “severe sepsis” and “systemic inflammatory response” were used for the first group; “strain echocardiography”, “global longitudinal strain” or “global circumferential strain” for the second group. The flow of references was managed with the Endnote X7 citation manager.

### Analysis of outcomes

The primary outcome was the relationship between GLS values of survivors and non-survivors from the cohort of patients with sepsis and/or septic shock and mortality at longest follow up, since most studies were expected to report mortality at several time intervals. From the studies reporting GLS values, we also assessed the difference in LVEF between surviving and non-surviving patients with sepsis and/or septic shock as the secondary outcome of our meta-analysis.

Five sensitivity analyses were planned: the first was conducted excluding studies with high risk of bias, the second using the “leave-one-out-at-a-time” approach, the third including data from studies providing data on patients later than 48 h after the diagnosis, the fourth including retrospective and case-control studies and the fifth grouping studies according to the software used for GLS assessment.

### Study selection and data extraction

Three investigators (FS, CC and AA) independently screened titles and abstracts produced by the search and identified potentially relevant articles. Full-text articles that were identified as relevant were then assessed against the eligibility criteria. Discrepancies were resolved by consensus and/or by involving other authors (MC, AVB and NF).

Two reviewers (FS and CC) independently extracted data from individual studies, contacted corresponding authors and entered information into a pre-designed data collection form. Data extracted from each study included the number of patients with sepsis and/or septic shock examined, the number of patients mechanically ventilated, the sequential organ failure assessment (SOFA) score and the longest follow-up mortality data, as shown in Table 2. All the authors conducted also an independent search on Medline to check for further evidence.

**Table 2:** Characteristics of included observational studies

Author, year population (number)	Echocardiography timing	GLS software and TTE views used	Data reported	SAPS SOFA APACHE	MV	Mortality	Longest follow up
Boissier, 2017 78 ICU patients with septic shock	TTE within 24 h of ICU admission	Philips’ Qlab 8.1 (Philips®) Ap: 4ch, 2ch	GLS and LVEF	60.1 ± 20.5 11.7 ± 3.4 -	84.6%	43.6%	Hospital
Chang, 2015 111 ICU patients with septic shock	TTE within 24 h of ICU admission	EchoPAC v. BT09 (GE®) Ap: 4ch, 2ch, 3ch	GLS and LVEF	- - 21 ± 8	65.8%	35.1%	Hospital
De Geer, 2014 50 ICU patients with septic shock	TTE within 24 h of ICU admission	EchoPac v. 112 (GE®) Ap: 4ch, 2ch, 3ch	GLS and LVEF	- 11 (9–12) -	84%	34%	90-day
Innocenti, 2016 56 ED patients with septic shock	TTE within 24 h if ICU admission	Philips’ Qlab 8.1 (Philips®) Ap: 4ch, 2ch	GLS and LVEF	- 6.3 ± 2.8 -	–	27.2%	28-day
Landesberg, 2014 106 ICU patients with severe sepsis or septic shock	TTE on ICU admission day or as soon as possible	Philips’ Qlab 8.1 (Philips®) Ap: 4ch, 2ch	GLS and LVEF	- - 21.61 ± 6.8	100%	39%	Hospital
Lanspa, 2017 298 ICU patients with severe sepsis or septic shock	TTE within 24 h of ICU admission	Image-Arena platform (TomTec®) Ap: 4ch	GLS	- 9 (6–12) 25 (18–23)	–	23%	28-day
Orde, 2014 60 adult patients with severe sepsis or septic shock	TTE within 24 h of meeting severe sepsis criteria	Syngo Velocity Vector Imaging (Siemens®) Ap: 4ch, 2ch, 3ch	GLS and LVEF	- 11 ± 4 -	65%	48%	180-day
Shahul, 2015* 35 ICU patients with sepsis and septic shock	TTE on admission and at 24 h post	cardiac perf. Analysis v1.1 (TomTec®) Ap: 4ch	GLS and LVEF	- 6 (2.1–9) 21.7 ± 6.2	69%	23.3%	30-day

Data on the number of patients on mechanical ventilation (MV) are reported, if available, at the time of echocardiographic assessment. Severity scores are provided according to the version reported by each study. Severity scores are reported according to the version of scoring adopted by the authors. Software used for global longitudinal strain (GLS) assessment are abbreviated for ease of reading

*ED* Emergency Department, *ICU* Intensive Care Unit, *LVEF* left ventricular ejection fraction, *TTE* trans-thoracic echocardiography, *Ap* apical view, *4ch* four-chamber view, *2ch* two-chamber view, *3ch* three-chamber view, *SAPS* simplified acute physiology score, *SOFA* sequential organ failure assessment, *APACHE* acute physiology and chronic health evaluation

*In this study we obtained data from the 35 patients with septic shock, while the remaining 15 patients with sepsis were excluded

### Quality assessment

Methodological design quality of the included observational studies was evaluated using the Newcastle-Ottawa scale (NOS) [20]. Briefly, the NOS appraises methodological quality in three domains: selection, comparability and outcome. Studies score points for each subset domain with a maximum of 9 points possible for assessing the quality of non-randomized studies in meta-analyses, and in particular they are classified as high-risk (1–3 points), intermediate-risk (4–5 points) or low-risk of bias (6–9 points).

### Statistical analysis

Mean values and standard deviation of the variables of interest were collected for the outcome analysis. If data were reported only as median and interquartile range or confidence interval, we followed the Cochrane’s recommendation to approximate the values of mean and standard deviation (SD) [21].

Continuous outcome differences were analyzed using an inverse variance model with a 95% confidence interval. Values are reported as standard mean difference (SMD), *P* values were two-tailed and considered significant if < 0.05. The presence of statistical heterogeneity was assessed using the X2 (Cochran *Q*) test. Heterogeneity was likely if *Q* > df (degrees of freedom), suggested and confirmed if *P* ≤ 0.10. Quantification of heterogeneity was performed using the *I*^2^ statistic. Values of 0–24.9%, 25.0–49.9%, 50.0–74.9% and > 75.0% were considered as none, low, moderate and high heterogeneity respectively [22]. If heterogeneity was quantified as low or above, a more conservative random model was used. Publication bias was investigated by inspecting the funnel plot. Meta-analysis was performed using the review manager (Revman) for MAC (Version 5.3. Copenhagen: The Nordic Cochrane Centre, The Cochrane Collaboration, 2014).

## Results

### Study selection

The literature search produced 49 titles on Medline and 33 on EMBASE. After eliminating duplicates, 64 titles were identified as potentially relevant. Abstracts were then appraised against inclusion criteria and full-text articles were retrieved for further analysis. Initially 39 articles were excluded because they were not relevant, and a further 18 studies were subsequently excluded for the following reasons: 4 focused on pediatric populations, 7 were animal studies, 4 were of a non-observational nature and 3 did not include a population with sepsis (see Additional file 1).

**Fig. 2:**
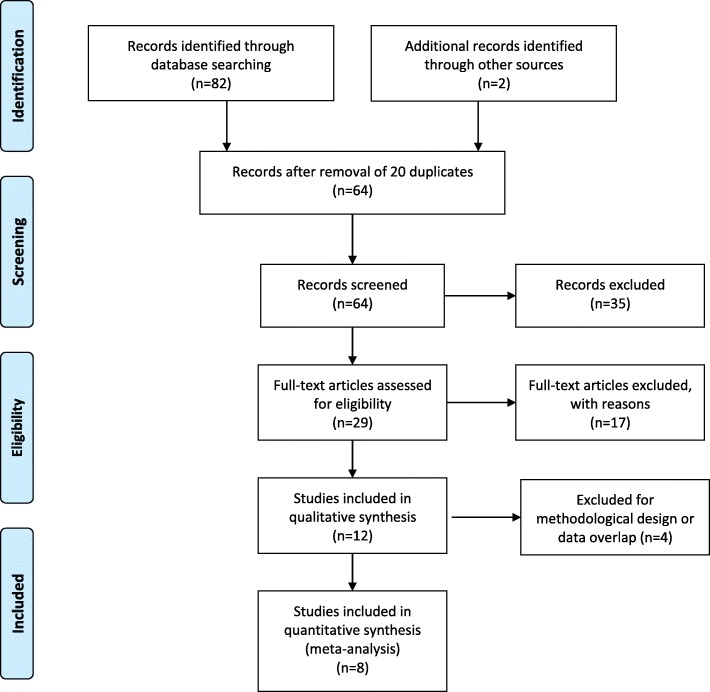
Preferred reporting items for systematic reviews and meta-analyses (PRISMA) flowchart of the study selection

We identified 12 studies as suitable for the meta-analysis; however most of the studies did not directly included GLS data in relation to survival. We contacted the corresponding authors and all of them were able to provide data on GLS (see “Acknowledgements”). One study [23] was excluded because it was a subset of another study published later [24]. Of the remaining 11 studies, we included only 8 for our primary analysis [24–31]. The other three studies were included in the sensitivity analyses only: one study because it reported GLS data collected beyond the first 48 h of admission [32], and two for their design (retrospective [33] and case-control [34]). Figure 2 shows the PRISMA flowchart of the systematic search and qualitative synthesis.

As shown in Table 2, which presents the studies’ characteristics, the timing of transthoracic echocardiogram assessment varied from less than 24 h to within 48 h of ICU admission, while the proportion of patients undergoing mechanical ventilation ranged from 65% to 100% of the population. The views used by each study to calculate GLS varied from a single apical view to the average of three apical views. Software from four different technology companies were used in the selected studies (Table 2).

### Primary outcome - GLS

**Fig. 3:**
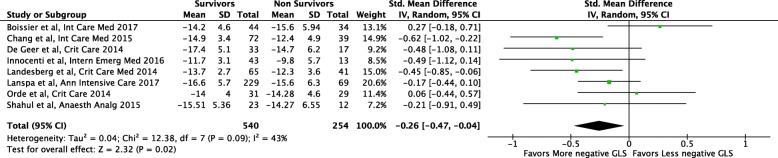
Comparison of global longitudinal strain (GLS) values between survivors and non-survivors among patients with severe sepsis and/or septic shock

Among the above studies, we collected GLS data on 794 patients, with an overall survival of 68% (*n* = 540). In the primary analysis including eight studies [24–31], survivors had more negative GLS values (better LV function) as compared with non-survivors (SMD − 0.26; 95% CI − 0.47, − 0.04; *p* = 0.02, Fig. 3) with low heterogeneity (*I*^2^ = 43%).

### Secondary outcome - LVEF (in studies reporting GLS values)

**Fig. 4:**
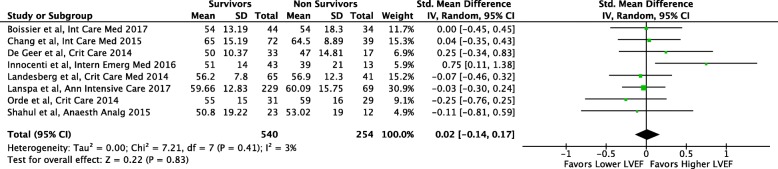
Comparison of left ventricular global ejection fraction (LVEF) values between survivors and non-survivors among patients with severe sepsis and/or septic shock, in studies also reporting global longitudinal strain (GLS)

The analysis of differences in LVEF between survivors and non-survivors included the same eight studies [24–31] and there were no differences in LVEF between groups (SMD 0.02; 95 % CI − 0.14, 0.17; *p* = 0.83, Fig. 4) with low heterogeneity (*I*^2^ = 3%).

### Sensitivity analyses

The first sensitivity analysis was performed including the study from Zaky et al. [32] (echocardiography conducted within the first week after ICU admission, 53 patients with sepsis and/or septic shock, in-hospital mortality). The inclusion of this study did not affect the results of both GLS and LVEF analyses (*p* = 0.03 and *p* = 0.64, respectively). The inclusion of the case-control study of Ng et al. [34] (echocardiography conducted within 48 h after ICU admission, 33 ICU patients with septic shock, 90-day mortality) reduced the strength of the association between GLS and mortality (*p* = 0.07) but left unchanged the results on LVEF (*p* = 0.83). Similarly, the inclusion of the retrospective study of Dalla et al. [33] (echocardiography conducted within 48 h after ICU admission, 48 patients with sepsis and/or septic shock, 30-day mortality) blunted the strength of the association between GLS and mortality (*p* = 0.08) but left unchanged the results on LVEF (*p* = 0.89).

The sensitivity analysis with the leave-one-out-at-a-time approach did not largely affect the results on GLS: removal of one of the four studies resulted in values between *p* = 0.05 [24, 27] and *p* = 0.08 [25, 26]. Removal of the other four confirmed *p* < 0.05 [28–31]. No differences were seen in the analysis on LVEF using the leave-one-out-at-a-time approach. The planned sensitivity analysis excluding the studies at high risk of bias according to the NOS was not performed because all the included studies scored between 7 and 9 points, thus qualifying as low risk of bias. The inspection of funnel plots confirmed no risk of publication bias. Software from four different technology companies were used for GLS analysis in the included studies; thus the analysis grouping studies  according to the software used was judged not feasible.

## Discussion

To our knowledge, this is the first meta-analysis investigating the role of STE in identifying patients with sepsis and/or septic shock at higher risk of mortality. We found a significant association between worse (less negative) GLS values and mortality, while the most commonly adopted conventional parameter - LVEF - was not associated with mortality when analyzing the results of the studies included in the meta-analysis, confirming previous findings [12].

Our findings are not entirely surprising since the value of GLS has been recognized in various settings. Strain imaging can detect subclinical myocardial dysfunction in experimental studies on septic animals. In a rabbit model, Li et al. showed that 2 h after injecting endotoxin, GLS declined before changes in LVEF were manifest [35]. Similar results have been reported in anesthetized pigs receiving infusions of *Escherichia coli*, where STE detected myocardial dysfunction before significant changes in LVEF and cardiac output [36].

In the clinical setting, GLS is helpful in the diagnosis and/or prognosis of early stages of heart failure [37], chemotherapy-related cardiotoxicity [38], hypertrophic cardiomyopathy [39], pregnancy-related myocardial dysfunction [40], cardiac amyloidosis [41] and subclinical cardiomyopathies in patients with chronic kidney disease [42]. A meta-analysis showed superior prognostic value of GLS as compared to LVEF in the prediction of major adverse cardiac events in a heterogeneous population with underlying cardiac conditions [43]. For such reasons, STE use has been included in updated American Society of Echocardiography and the European Association of Cardiovascular Imaging guidelines [44]. All together with its prognostic value, GLS has been shown to have good reproducibility and non-significant intra-observer error, in many cases outperforming most conventional echocardiographic parameters, including LVEF, although small but significant inter-vendor differences still persist [45].

The aforementioned guidelines suggest normal values of peak GLS in the range of − 20% [44]. In our meta-analysis none of the reported mean GLS values fell in this range, reinforcing the fact that a degree of systolic impairment may be present in the majority of patients with sepsis and/or septic shock, both in survivors and non-survivors. Therefore, GLS could be considered a better surrogate of intrinsic LV myocardial function contrary to LVEF. One animal study showed 100% of myocardial depression as assessed via the pressure-volume loop (gold standard method) [46], but this finding differs from other studies [47, 48], leaving uncertainties in this field. Nonetheless, GLS may become a very useful parameter in the early evaluation of septic cardiomyopathy. For instance, Boissier et al. elegantly showed impaired values of GLS in the first 24 h in patients developing secondary LV hypo-kinesia (defined as reduced LVEF on day 2 or day 3 after a normal value of LVEF on the first day in the ICU), confirming higher sensitivity of GLS in detecting sub-clinical cardiomyopathy. Moreover, in this study even patients with normal LVEF had mean GLS values worse than − 20%, highlighting the ability of GLS in detecting myocardial impairment not shown by LVEF. Moreover, only a few patients had good GLS values and all of them had normal LVEF [31].

Importantly, GLS values are different in other populations of critically ill patients, as shown by Dalla et al. [33] who reported a prevalence of depressed longitudinal LV function as high as 50% in patients with septic shock, while the prevalence was below 9% in controls (trauma patients without septic shock) [33]. Similarly, Ng et al. [34] observed worse GLS values in patients with septic shock as compared with those with sepsis only (− 14.5% vs − 18.3%, respectively) and significantly improved strain after recovery from septic shock in patients weaned off vasopressors within 72 h. However, Ng et al. [34] also demonstrated some improvement (not statistically significant) in GLS in a smaller group of patients remaining vasopressor-dependent [49]. On the contrary, another study in patients with septic shock found unchanged GLS over time despite normalization in LVEF and cardiac biomarkers and clinical recovery [27].

The use of STE may possibly overcome some limitations of LVEF, in relation to variability of loading conditions and geometric assumptions, indeed, as LVEF is a direct measure of the change in blood pool volume and thus dependent on volume and pressure load on the myocardium [50, 51], with systolic function increasing during vasodilatation and decreasing in states of vasoconstriction [51, 52]. It has been claimed that GLS is a reliable, reproducible, and sensitive modality for assessing cardiac systolic function [43, 53, 54]; however, it should be kept in mind that GLS is also dependent on LV loading conditions, especially afterload changes, with longitudinal fibers suffering from higher wall stress due to their orientation. This assumption is confirmed by animal [55] and clinical studies [56–59]. A recent study by Nafati et al. demonstrated that in a heterogeneous population of preload-dependent critically ill patients, GLS is significantly affected (more negative values indicating improved LV function) after fluid resuscitation [60]. In critically ill patients with sepsis and/or septic shock it is possible that after the initial fluid resuscitation, afterload changes play a more important role than preload, particularly in terms of outcome. For instance, in patients with aortic stenosis with varying afterload, GLS was shown to be sensitive to these changes, while LVEF was not [61, 62]. It may be that this is also reflected in the value of GLS to predict mortality. Certainly, more research is warranted to understand the variation in GLS according to preload and afterload under stable hemodynamic and respiratory support in critically ill patients. Of course, LVEF variations are also related to changes in afterload more than in intrinsic myocardial contractility [14], and in this regard Vieillard-Baron et al. repeatedly confirmed the variability in the incidence of systolic dysfunction as evaluated by LVEF at different time points after the onset of septic shock [11, 51]. An independent association between hyperdynamic conditions (LVEF > 70%) and mortality was identified in a large retrospective cohort study of critically ill patients [63]. A further limitation in the estimation of systolic function by means of LVEF is the geometric assumptions. Wall motion measurements evaluating displacement cannot differentiate between active or passive movement of a myocardial segment with inward systolic movements dragging diseased segments, and thus the resulting LVEF is relatively unaffected by subtle systolic changes [64]. On the contrary, deformation analyses (i.e. GLS) allow discrimination between active and passive myocardial tissue movement and are less affected by measurement errors because they avoid geometric assumptions [65]. It is possible that a reduction in longitudinal strain is compensated by other factors and LVEF remains normal for such mechanisms. A recent study confirmed the hypothesis that strain better reflects systolic function as compared with LVEF, especially in patients with preserved LVEF, where the authors showed a flatter slope for GLS/LVEF correlation (slope − 0.6) as compared with patients with decreased LVEF (slope − 1.6). Such findings indicate that LVEF may be unaffected if a reduction in GLS is compensated by a change in other parameters (increase in circumferential strain and/or increase in LV wall thickness and/or reduction in LV end-diastolic volume), and that GLS can decrease earlier before effects on LVEF are manifest [66].

The use of GLS probably offers advantages over assessment of the systolic pulsed wave (s’) obtained with tissue Doppler imaging (TDI). The s’ TDI of the base of LV wall is another index of longitudinal LV systolic function and has been validated against LVEF in patients with cardiac disease [67]. In critically ill patients, TDI has been predominantly used in the assessment of LV diastolic function [9], but there is growing literature on s’ TDI [25, 26]. However, s’ provides an angle-dependent, unidirectional (longitudinal) and unidimensional (one LV segment) assessment of myocardial motion, thus being exposed to underestimation or overestimation (regional wall motion abnormalities, tethering, etc.). In this context, GLS has advantages over TDI, likewise assessing all the LV segments and being less angle-dependent [68, 69], but on the other hand better imaging definition is required compared with TDI [70]. Possible M-mode surrogates of GLS may be longitudinal wall fractional shortening, the curved anatomical M-mode fractional shortening and the mitral annulus systolic plane excursion. Such parameters have recently been shown to correlate strongly with GLS, with longitudinal wall fractional shortening being the best unbiased strain predictor. Given the simplicity of M-mode measurements, this may have significant clinical and practical implications for future critical care echocardiography investigations [71].

From a clinical perspective, obtaining a more accurate identification of underlying septic cardiomyopathy has not only prognostic but also therapeutic value. For instance, tachycardia is one of the hallmarks of sepsis and it is associated with worse clinical outcomes in critically ill patients [72]. However, it is still difficult to distinguish between tachycardia as an adaptive response to low preload conditions and tachycardia related to a persistent hyperadrenergic state (in turn responsible for septic cardiomyopathy). Lanspa et al. identified an association between impaired GLS and tachycardia [28]. Despite this, controversy remains surrounding the use of beta blocker therapy for treatment of tachycardic patients with sepsis and/or septic shock [73, 74], and it is advisable that future studies on beta blockade in sepsis evaluate LVSD by STE for the identification of patients who might benefit from pharmacological heart rate control.

Furthermore, with the wider use of echocardiography we believe that RV function measured with STE should be a focus of future research. The RV has a preponderance of longitudinal fibers and can be assessed with longitudinal strain [75] and there is growing evidence with regard to the prognostic value of RV strain, for instance in patients with pulmonary hypertension [76]. The utility of RV strain in sepsis has not been assessed in our meta-analysis because of the small number of available studies, but interesting findings have been reported by Orde et al., where higher RV strain was identified in sepsis survivors despite similar values of LV strain [29]. However, RV function - more than LV - is affected not only by preload but also by mechanical ventilation, making its assessment challenging in mechanically ventilated patients with septic shock.

### Limitations

Our meta-analysis has the main limitation of exploring an association between values of GLS and mortality in patients with severe sepsis and/or septic shock and GLS values that were directly reported or collected by contacting corresponding authors, but that are not adjusted for confounders by regression/multivariate analyses. It would have been valuable to adjust these values for patient’s severity score, presence and mode of mechanical ventilation, dose of vasoconstrictors and hemodynamic conditions and fluid balance, etc. Unfortunately, this is not feasible without accurate access to individual patient data from all studies, and it remains the main limitation of the present meta-analysis. Moreover, with regards to the GLS, half of the sensitivity analyses moved the *p* value between 0.05 and 0.08, while no changes were noted on LVEF which continued not to be associated with sepsis outcome.

We found only two papers also reporting values of circumferential and/or radial strain [26, 30], therefore these values were not considered for analysis. Nonetheless, sub-endocardial myocardial fibers are oriented longitudinally and these fibers are especially sensitive to ischemia and increased wall stress [77], thus GLS could be reliably considered as a marker of myocardial dysfunction in sepsis.

There are some clinical limitations in the introduction of GLS in clinical practice. An initial one was posed by inter-vendor differences in software algorithms, which makes normal values difficult to standardize due to biases among vendors. Software uses information gathered from different myocardial layers, endocardial (E-GLS) and mid-wall (M-GLS) for the calculation of GLS. Literature suggests that there is no difference in terms of robustness between E-GLS and M-GLS across vendors [78] and a recent study showed good reproducibility of GLS measurements, which in many cases was superior to other conventional echocardiographic measurements [45]. We were unable to analyze subgroups of studies according to the software used for GLS assessment as software from four different technology companies was used for GLS quantification in the eight studies included. With technological advances, expert calls for concordance on vendor strain software analysis, and with more widespread use of GLS, we anticipate that STE may become a routine measurement in the future. In the past, GLS analysis was mainly performed as an off-line calculation using remote workstations rather than being a bedside application. Currently, after appropriate training, doctors may assess GLS in about 10 min per patient, and new advanced echocardiographic machines have embedded software packages for real-time GLS measurement, making such approaches applicable at the bedside. Strain assessed with STE measures both regional and global functions, assessing the function of sub-endocardial longitudinal fibers of all 17 myocardial segments, and has been validated as the most consistently reproducible measurement [79]. Although global strain values may not entirely reflect segmental changes, septic cardiomyopathy is more of a general process mediated by a hyper-adrenergic state [80] combined with tissue inflammation [81], sarcolemmal permeability [82], free radicals [83] and mitochondrial dysfunction [84]; therefore, the limitation of global assessment is likely to be clinically irrelevant in this group of patients.

However, the frame rate of STE is limited to the relatively low frame rate of the B-mode. When the frame rate is too low, the tracking quality is reduced due to frame-to-frame decorrelation. This can often be a problem if the heart rate is high, which happens in most patients with sepsis. Strain measurement does not only require training and time at the bedside, but needs high-quality images. Studies in the non-critically ill report 7–9% suboptimal image quality for STE analysis [85, 86], and one study in patients with sepsis reported slightly higher incidence (13%) [29]; however, a more recent study on STE reported the feasibility of at least one GLS measurement during the first 3 days in the ICU in up to 59% of the population (*n* = 78/132) [31]. It should be noted that the feasibility of GLS assessment may depend on the methodology of acquisition and on patient-related factors. In the first case, feasibility may become lower if the clinicians decide to acquire GLS averaging three apical views (in our meta-analysis this was done only in three out of eight studies). From the patient’s perspective, chronic obstructive pulmonary disease could be one of the main factors associated with inability to obtain echocardiographic images suitable for quantitative assessments, as shown by one study for both LVEF and GLS [23]. Although echocardiography imaging in the critically ill can be difficult, ICU physicians keen to implement echocardiography should keep in mind that STE still remains feasible in a large number of patients!

## Conclusions

Worse values of global longitudinal strain are associated with higher mortality in patients with severe sepsis or septic shock, while such an association is not valid for left ventricular ejection fraction. More research is warranted to elucidate such an association, which could be related to the ability of speckle-tracking echocardiography in demonstrating underlying intrinsic myocardial disease as opposed to left ventricular ejection fraction.

## Additional file

Additional file 1: Newcastle-Ottawa scale for assessment of the quality of included cohort studies. Each asterisk represents fulfilment of the acceptable criteria within each subsection. (DOCX 17 kb)

## Abbreviations

GLS: Global longitudinal strain
LV: Left ventricular
LVEF: Left ventricular ejection fraction
LVSD: Left ventricular systolic dysfunction
NOS: Newcastle-Ottawa scale
PICOS: Patient, population or problem, intervention, comparison, outcomes and study design (or setting)
PRISMA: Preferred reporting items for systematic reviews and meta-analyses
SOFA: Sequential organ failure assessment
STE: Speckle-tracking echocardiography
TDI: Tissue Doppler imaging
