# Supplementary material for: Left ventricular systolic function evaluated by strain echocardiography and relationship with mortality in patients with severe sepsis or septic shock: a systematic review and meta-analysis
Source: Crit Care. 2018 Aug 4;22:183. doi: 10.1186/s13054-018-2113-y (PMC6091069; doi:10.1186/s13054-018-2113-y)
Supplement: Supplementary file 1 — Newcastle-Ottawa scale for assessment of the quality of included cohort studies. Each asterisk represents fulfilment of the acceptable criteria within each subsection. (DOCX 17 kb) [file 13054_2018_2113_MOESM1_ESM.docx]

Additional file 1. Newcastle-Ottawa scale for assessment of quality of included cohort studies. Each asterisk represents fulfillment of the acceptable criteria within each subsection

| **QUALITY ASSESSMENT CRITERIA** | **ACCEPTABLE(*)** | **Landesberg 2014** | **Orde 2014** | **De Geer 2015** | **Chang 2015** | **Shahul 2015** | **Innocenti 2016** | **Lanspa 2017** | **BoiSsier 2017** |
| --- | --- | --- | --- | --- | --- | --- | --- | --- | --- |
| **Selection** |  |  |  |  |  |  |  |  |  |
| Representative of exposed cohort | Representative of average adult patient presenting with severe sepsis/septic shock | * | * | * | * | * | * | * | * |
| Selection of the non-exposed cohort | Drawn from same community as exposed cohort | * | - | * | * | * | * | * | * |
| Ascertainment of exposure | Secured records/electronic database | * | - | * | * | - | * | * | * |
| Demonstration that outcome of interest was not present at start of study | Only incident cases of sepsis | * | * | * | * | * | * | * | * |
| **Comparability** |  |  |  |  |  |  |  |  |  |
| Study controls of age/sex | yes | * | * | * | * | * | * | * | * |
| Study controls for at least 3 additional factors | Ventilation, time of assessment, known heart disease, ICU severity scores(APACHE) | * | * | * | * | * | * | * | * |
| **Outcome** |  |  |  |  |  |  |  |  |  |
| Assessment | Independent blind assessment/record linkage | * | * | - | * | - | - | * | - |
| Was follow up enough for outcome to occur | Follow up until hospital discharge | * | * | * | * | * | * | * | * |
| Adequacy of follow up for cohort | Complete follow up ,or subjects lost to follow up unlikely to introduce bias | * | * | * | * | * | * | * | * |
| **OVERALL QUALITY SCORE** |  | 9 | 7 | 8 | 9 | 7 | 8 | 9 | 8 |
